# Recent Advances in Real-Time Time-Dependent Density Functional Theory Simulations of Plasmonic Nanostructures and Plasmonic Photocatalysis

**DOI:** 10.1021/acsnanoscienceau.2c00061

**Published:** 2023-05-19

**Authors:** Connor J. Herring, Matthew M. Montemore

**Affiliations:** Department of Chemical and Biomolecular Engineering, Tulane University, New Orleans, Louisiana 70115, United States

**Keywords:** Plasmonic catalysis, Time-dependent density functional theory, Metal nanoparticles, Electric fields, Plasmonics

## Abstract

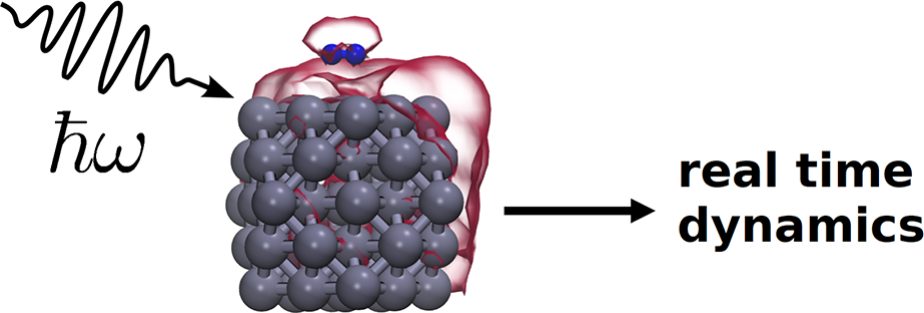

Plasmonic catalysis provides a possible means for driving chemical reactions under relatively mild conditions. Rational design of these systems is impeded by the difficulty in understanding the electron dynamics and their interplay with reactions. Real-time, time-dependent density functional theory (RT-TDDFT) can provide dynamic information on excited states in plasmonic systems, including those relevant to plasmonic catalysis, at time scales and length scales that are otherwise out of reach of many experimental techniques. Here, we discuss previous RT-TDDFT studies of plasmonic systems, focusing on recent work that gains insight into plasmonic catalysis. These studies provide insight into plasmon dynamics, including size effects and the role of specific electronic states. Further, these studies provide significant insight into mechanisms underlying plasmonic catalysis, showing the importance of charge transfer between metal and adsorbate states, as well as local field enhancement, in different systems.

## Introduction

Photocatalysis, in which light is used to drive a chemical reaction, has been extensively studied as a means of alternative energy harvesting. While research on photocatalysis has largely been focused on semiconductors, certain plasmonic metal nanostructures have recently demonstrated superior photocatalytic capability for a variety of reactions.^1^ These systems offer certain advantages over thermal heterogeneous catalysts for not only their ability to harness light but also their ability to catalyze reactions at mild reaction conditions.^2^ More broadly, plasmonic metal nanostructures have extensive applications in chemical or biological sensing, spectroscopy, and nanoscale control of light.^3,4^

Understanding the behavior of plasmons and their interactions with adsorbing species is a critical issue for rational design of plasmonic catalysts. Plasmon-mediated reactions are thought to be related to localized surface plasmon resonances (LSPRs), which refer to strong, collective oscillations in a metal’s free electron density. However, direct observation of LSPRs at short length scales and time scales is difficult. As a consequence, there has been significant disagreement on the mechanisms underlying plasmonic catalysis. For example, both hot electrons and near-field enhancements have been proposed and experimentally supported for the plasmon-promoted dissociation of O_2_ on Ag.^5−7^ Other possible excitation mechanisms include sequential charge transfer between metal and molecular states and local heating.^7^

Real-time time-dependent density functional theory (RT-TDDFT)^8−12^ has been used to study plasmons and other excitations in nanomaterials due to its ability to give clear insight into dynamic processes related to excited states. The lower computational cost of generalized gradient approximation (GGA) functionals implemented in RT-TDDFT compared to more high-level methods, coupled with its demonstrated agreement with these approaches for certain properties (such as the system dipole moment),^13^ has made RT-TDDFT a quite useful tool in a wide variety of plasmonic studies. Higher level methods are still expected to produce more accurate results than the commonly applied RT-TDDFT approaches but the trade-off for computational expense makes RT-TDDFT a logical choice in many cases.

In this Perspective, we discuss recent findings that use RT-TDDFT to study plasmons, focusing where possible on those that study plasmonic catalysis. In general, these studies have used RT-TDDFT as a method to visualize and understand plasmon dynamics, to elucidate system size and composition effects on plasmons, and to gain insight into mechanistic questions regarding plasmonic catalysis.

## Methods Used in RT-TDDFT Plasmon Studies

RT-TDDFT differs from frequency-space TDDFT by utilizing explicit time propagation of the Kohn–Sham eigenstates. This yields the nonadiabatic electronic density at each time step in the simulation and allows for applications in nonlinear regimes, such as strong electric fields. This also allows studies of atomic motion in reaction to these fields. Conversely, linear response TDDFT (LR-TDDFT) involves the use of the Casida equations to calculate the excitation spectrum; this can be very demanding for large systems. Specifically, LR-TDDFT requires calculation of a large number of unoccupied orbitals and the transitions between occupied and unoccupied orbitals. The number of unoccupied orbitals determines the size of the matrix equations needed, and this leads to the high computational expense for large systems.^14,15^ LR-TDDFT has been demonstrated to be effective when applied to systems with molecular orbitals that are not highly delocalized.^9,16^ In these cases, LR-TDDFT has seen substantial and rapid success for calculating excited states. A number of studies discussed in this Perspective compare RT- and LR-calculated excited states. Overall, good agreement in absorption spectra has been seen between RT-TDDFT and LR-TDDFT, indicating that the Fourier-transform based method for the extraction of excitation information works well.^13,15,17−19^ However, once outside of the weak field regime the comparison between RT and LR methods becomes infeasible as nonlinear effects are not captured by LR-TDDFT.^20^ Additionally, the higher computational expense of LR-TDDFT (due to the large matrix size) makes obtaining spectra with many states difficult (compared to the single Fourier transform required for RT-TDDFT). For these reasons, comparisons of the two methods have been limited to relatively weak electric fields and relatively small systems. While the RT approach makes accessing the high-field regime feasible, it is possible that the adiabatic approximation (discussed later in this section) may not be a valid assumption as the electron density evolves significantly away from the ground state.

RT-TDDFT studies of plasmonic systems often begin by calculating the absorption spectra of the systems being studied. The absorption spectrum is found by first exciting the system with an electric field in the form of a weak δ-kick at *t* = 0, after which the system evolves without any additional perturbation (per the Yabana and Bertsch method).^21^ This gives the time-dependent dipole moment, which is then Fourier transformed to give the absorption spectrum. The maximum peak of the resulting absorption spectrum corresponds to the plasmon mode, and this plasmonic frequency is generally used for subsequent excited state calculations of the system being studied, i.e., as the frequency (ω) in eq 1.

For observation of the plasmon, a laser-like electric field composed of a sinusoidal component with a Gaussian envelope is generally used to excite the system. This allows the system to initialize into the ground state and then become excited as the field gains non-negligible amplitude. For example,
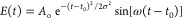
1where *A*_o_, *t*_0_, σ, and ω are the maximum amplitude, center, width, and frequency, respectively.^13,22−24^ Generally, a quite strong field is used to excite the system, which allows clear observations of plasmon dynamics and any subsequent chemical reactions on the short time scales that are feasible to RT-TDDFT simulations (generally <1 ps). Furthermore, it is common for the pulse to be polarized along one axis to observe any potential effects of field polarization on plasmon generation or interactions.

More efficient real-time methods such as the tight-binding (RT-TDDFTB) approach have been utilized for studying relatively large plasmonic systems over longer time scales.^25−27^ In return for sacrificing some level of quantum mechanical accuracy, RT-TDDFTB allows for the investigation of electronic dynamics of systems with several hundred atoms and at time scales into the picosecond regime. Comparison of this method to RT-TDDFT and higher levels of theory, particularly within the field of plasmonics, is still an avenue to be explored.

A wide variety of functionals and approaches have been implemented in RT-TDDFT for describing electronic structure of plasmonic materials (discussed throughout this Perspective). Most of these approaches make a number of cost-saving approximations. Other approaches to simulating excited states, such as correlated wave function theories, have been used to study plasmonic systems for a variety of applications.^28−32^ These studies are outside the scope of this Perspective as they do not utilize RT-TDDFT but are examples of potentially more accurate methods which can be used to compare the accuracy of future RT-TDDFT studies. There are also several methodologies used to treat nuclear motion when studying excited-state dynamics in nanosystems, such as Ehrenfest dynamics,^33^ Liouville–von Neumann molecular dynamics with real-time tight binding,^19,34^ surface hopping,^35^ and a number of techniques based on surface hopping.^36−39^ There are trade-offs between these various methods, and for brevity we do not provide a comprehensive discussion of these trade-offs. Ehrenfest dynamics in particular excels in providing dynamic information at a relatively reasonable computational cost. In addition to the dynamic insights available from these simulations, the reasonable computational cost allows studies of more realistic systems than is possible using many other techniques. However, depending on its implementation, Ehrenfest can lead to qualitatively different dynamic outcomes (even when the same approximate functional is used) and can diverge from LR or “exact” solutions under certain conditions.^40^ Further, Ehrenfest dynamics does not obey detailed balance and will perform poorly when a system has two quite different possible states both with high probabilities.^8^ Ehrenfest dynamics is generally expected to perform well over short time scales in systems with many similar potential energy surfaces, as is the case in many solid-state systems and nanomaterials.

There are open questions about some of the approximations used in most RT-TDDFT calculations. Perhaps most notable is the adiabatic approximation, which nearly all practical implementations of RT-TDDFT rely upon. In this approximation, only the instantaneous electron density is used with a ground state functional, so there is no history or “memory” included in the exchange-correlation functional. Ideally, the exchange-correlation potential would be a functional of the time-dependent electron density in addition to the initial interacting wave function and noninteracting Kohn–Sham orbitals.^11^ The lack of this history dependence in the adiabatic approximation has been found to lead to peak shifting and other nonphysical effects.^20,41,42^ While these issues are seen to arise in small molecules such as H_2_, it is not clear whether these issues will arise in a substantive way in plasmonic systems, which have quite different excitation properties in many respects. In particular, the studies presented in this Perspective do not definitively establish whether these types of issues are present for RT-TDDFT studies of larger plasmonic systems. However, the general agreement between LR and RT methods in the weak field regime across a number of different systems is at least encouraging that RT-TDDFT results are reasonable. Precisely how reliable various results are as the system is perturbed far from the ground state is still an open question and should be kept in mind when evaluating the conclusions drawn in these studies. Further approximations utilized by RT-TDDFT include GGA functionals designed for the ground state, in addition to physical simplifications made for the systems studied such as small nanoparticles and short time scales. Some work has been done to compare GGA functionals to hybrid and long-range corrected (LC) functionals for excited states.^13^ In this study, reasonable agreement was seen between GGA and LC calculated absorption spectra. Generally, until higher level methods are able to perform dynamic simulations on relatively large systems, it is difficult to quantify the level of approximation for particular systems. As is the case for most DFT calculations, the calculations almost certainly do not give the very high accuracy that is possible for many smaller systems using high-level wave function methods. However, the results of previous RT-TDDFT calculations on the kinds of systems discussed here are generally in qualitative agreement with experiments where available and seem to be physically reasonable.

From the perspective of computational expense, one of the primary difficulties for RT-TDDFT is the short time step required to capture electron dynamics, often on the scale of tens of attoseconds. In contrast, ground-state DFT-based molecular dynamics simulations (which do not include the propagation of electrons) often use a time step of roughly 0.5 to 1 fs. For this reason, molecular dynamics with RT-TDDFT is generally significantly more computationally intensive for a given simulation time than ground-state molecular dynamics using DFT. It is believed that larger time steps can be used in RT-TDDFT via an optimal gauge choice in the Schrödinger dynamics, and while there has been some work addressing this issue,^43^ this is an area of active research and techniques for allowing larger time steps have not been implemented in all codes.

## Plasmon Dynamics and Properties in Metal Systems

RT-TDDFT allows direct observation of plasmon dynamics at the atomic scale in a way that is difficult to access using other techniques. For example, the RT approach can be used in systems with many excited states and for applying strong electric fields. Thus, RT-TDDFT can give insight into fundamental and important questions related to plasmon dynamics, such as which electronic metal states are important for plasmon generation, what role do d electrons play in these processes, how does the generated plasmon behave over time, what effect do multiple nanoparticles have on one another, and how can chain-like nanoparticle arrangements be used for the nanoscale control of light?

**Figure 1:**
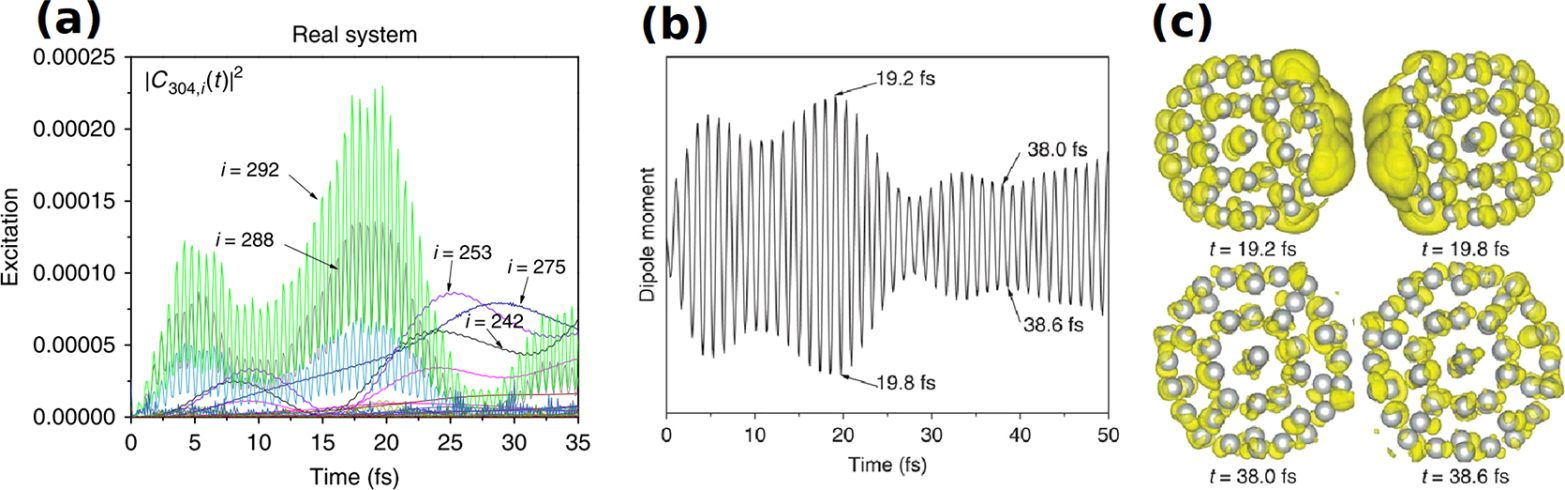
(a) The transition from the *i*th state to the LUMO (state 304). Two distinct transition types were observed: rapidly oscillating and smoothly varying. (b) The dipole moment over time for an icosahedral Ag_55_ structure. (c) The induced charge density in the cluster at four time points. Charge initially sloshes back and forth on the nanoparticle surface but begins to delocalize as time evolves and the dipole moment amplitude diminishes. Panels a–c are the result of applying an external laser with a frequency equal to the calculated Ag_55_ absorption peak, ω_p_ = 3.6 eV. Adapted with permission under a Creative Commons License from ref (44). Copyright 2015 Springer Nature.

To provide insight and to separate out the contributions of plasmonic and single-particle excitations in a metal nanoparticle, plasmon generation in icosahedral Ag_55_ was studied (Figure 1).^44^ Two distinct excitation types were observed: oscillatory and smoothly varying. The oscillatory excitations corresponded to the plasmon mode and the rest to single-particle excitations from hot carriers (discussed further in the next paragraph). Hot carriers originated from low energy d-states and were excited to eigenstates around the Fermi energy.^44^ Further, the hot carrier generation was slowed by adding one electron to the Ag structure due to Ag_55_^–^ being a closed shell system. Lastly, it was concluded that electron–phonon interactions have minor effects in the nanocluster.

Distinguishing plasmonic excitations from single-particle excitations can be difficult as these two types of excitations often have overlapping resonance frequencies. In the case of Ag_55_ discussed above, this distinction was determined by comparing the energies of each eigenstate (ω_*i*_) to the energies of the LUMO (ω_LUMO_) and the plasmon (ω_p_). Single-particle transitions can only be excited if they have nearly resonant frequencies (ω_LUMO_ – ω_*i*_ ≈ ω_p_), and the eigenstate energies of the smoothly varying excitations were all nearly resonant while the rapidly oscillating transitions were not. Further, the Fourier transform of the two types of transitions revealed that the slowly varying curves had just one peak around ω = 0 while the rapidly oscillating curves had two peaks. The wave equation for the smoothly varying oscillations could be captured by single-particle time-dependent perturbation theory while the rapid oscillations could not, which is consistent with the notion that the smoothly varying oscillations represent single-particle excitations while the rapid oscillations represent collective or plasmonic oscillations. A separate but similar approach for distinguishing excitation types visualized the process with transition contribution maps, plotting the transitions as a function of the energies of the corresponding occupied (ϵ_o_) and unoccupied (ϵ_u_) states. These plots helped to show that transitions whose energy difference (ϵ_o_ – ϵ_u_) approximately equaled the applied frequency corresponded to single-particle transitions, while off-resonant energy differences were plasmonic.^14^

Plasmon resonances decay quickly once irradiation ceases, with the LSPR lifetime generally being experimentally measured at ≤10 fs.^3^ Since many plasmonic applications are aided by slower dampening, it is of interest to better understand plasmon decay. These decay mechanisms were studied in a bare tetrahedral Ag_8_ nanocluster using RT-TDDFT.^18^ The variation in off-diagonal density matrix elements provided insight into energy transfer during the simulation. It was found that the one-photon-allowed transitions undergo ultrafast decay into high energy transitions such as two-photon-allowed transitions.

In addition to three-dimensional structures such as nanoparticles, many studies have looked at linear metallic chains as they may be considered quasi-one-dimensional and are thought to display unique spectral properties.^45^ Linear chains of Na_20_ and Ag_37_, as well as icosahedral Na_55_^+^, were studied using RT-TDDFT.^15^ Ag_37_ nanorods showed that the primary contributions to the LSPR mode come from the ends of the rod, while reconstructed modes at lower energies are localized around Ag atoms, indicating strong contributions from d electrons. Finally, the absorption spectrum of icosahedral Na_55_^+^ showed that the induced density comes primarily from the cluster surface.

While quasi-one-dimensional and three-dimensional structures represent the bulk of the work done to date, two-dimensional materials are an interesting next application of RT-TDDFT methods. In this area, MXenes have been investigated, specifically a monolayered Al sheet on top of a Ti_3_C_2_F MXene.^46^ Both the Al sheet and the Ti_3_C_2_F MXene showed significantly reduced absorption when the field was oriented normal to the surfaces and the plasmonic features largely disappeared. The disappearance of plasmonic behavior in the *z* direction was likely due to quantum confinement effects. In the hybrid structure, electron accumulation was seen on the outer surfaces composed of F atoms while electron depletion was observed in regions around Ti atoms, which is in agreement with the larger Pauling electronegativity of F.

As mentioned previously, a handful of studies have begun to extend the accessible time scale and system size through the use of the tight-binding approach (RT-TDDFTB). These studies have examined Ag_14_ dimers,^27^ chains of up to eight Ag_55_ nanoparticles,^26^ and chains of up to four Na_55_ nanoparticles^25^ (separated by distances as short as 0.5 and as long as 50 Å). At very short interparticle distances, quantum mechanical effects such as electron tunneling and charge transfer plasmons become relevant, which the tight-binding approach is able to capture. These studies have found both that electronic excitation transfer can occur at distances significantly longer than the cutoff limit imposed by Förster resonance energy transfer-based approaches and that the efficiency of excitation transfer increases as the interparticle distance decreases up until a certain critical distance. At this point excitation transfer starts to become less efficient due to back-transfer effects between nanoparticles. This back-transfer is believed to be due to beating between the applied external laser acting on nanoparticles in a chain-like arrangement.

These studies show the detailed insight RT-TDDFT can give into plasmon dynamics at short time scales and length scales. In most cases, spatial oscillations in the charge density are observed while external the field has a significant amplitude, and these oscillations quickly cease after the field dies down. Several studies show that d electrons often play an important role in plasmonic processes and that it is possible within the RT scheme to decompose excited states into contributions from excitations of different types (e.g., plasmonic vs single-particle). Further, the electric field polarization can impact how a plasmon is generated and behaves, at least in two-dimensional materials or when quantum confinement effects are prevalent. These quantum effects (discussed further in the next section on system size effects) can play a significant role at the sub-nanometer scale and should be considered when dealing with small systems, especially those consisting of multiple nanoparticles.

## System Size Effects on LSPR

There has been significant effort in the field of plasmonics toward understanding how changes in nanomaterial size and shape affect the plasmonic behavior. RT-TDDFT allows for study of these effects in a way that includes the atomic-scale structure, quantum confinement effects, and changes over time.

Quantum effects such as the quantization of electron energy levels and d-electron screening have been found to be relevant in nanoparticles as large as 10 nm.^47,48^ Above this size the plasmon resonance of nanoparticles can be described reasonably well by classical dynamics like Mie theory.^49^ Additionally, the local density approximation (LDA) and GGA are both known to overestimate the energy of the d-electron band and thus d-electron screening.^50^ Since the d-band is important for describing plasmonic properties, especially in the small “quantum size” regime, it is necessary to accurately characterize the properties of the d electrons. In the context of RT-TDDFT this means that a method that adequately includes correlation effects (ideally long-range corrections)^51^ for the system under study should be used. Doing so has been found to produce better asymptotic behavior than LDA and GGA^52^ and to predict a more accurate d-band for Ag.^53^

**Figure 2:**
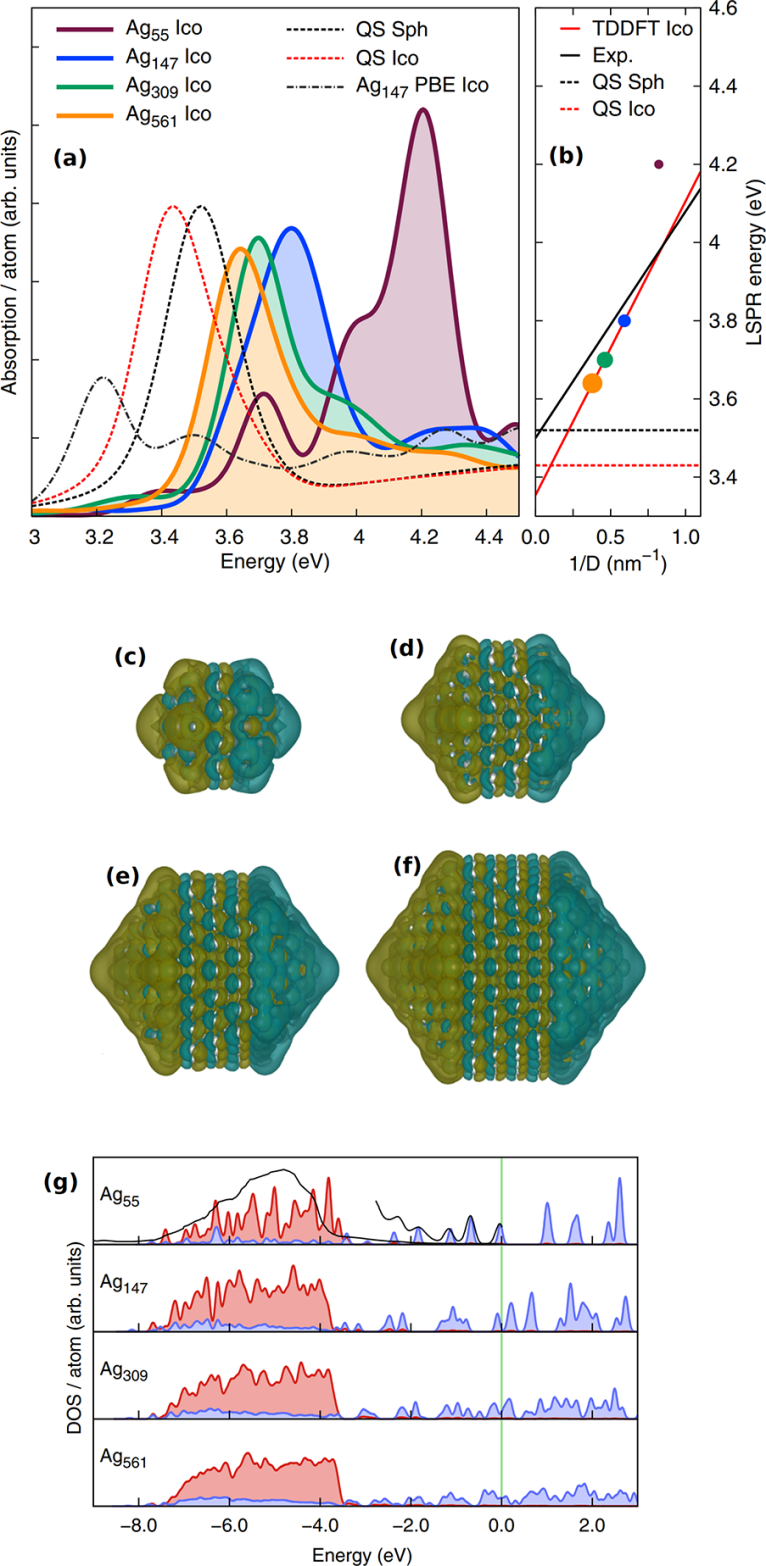
(a) Absorption spectra for Ag nanoparticles normalized by the number of atoms. The spectra for Ag_55_, Ag_147_, Ag_309_, and Ag_561_ were calculated with the GLLB-SC functional via TDDFT and compared to the PBE-calculated spectrum of Ag_147_. The quasistatic finite difference time domain (QSFDTD)^54^ spectra are also shown for spherical (Sph) and icosahedral (Ico) Ag nanoparticles. The dashed horizontal lines represent classical limits for the size invariant implementation of the QSFDTD method^55^ for spherical and icosahedral nanoparticles. (b) LSPR energy (circular dots) of icosahedral Ag nanoparticles as a function of inverse diameter. (c–f) The induced electron densities of Ag_55_, Ag_147_, Ag_309_, and Ag_561_. (g) Density of states of Ag_55_, Ag_147_, Ag_309_, and Ag_561_, separating out the d band (red) and sp band (blue).^50^ Adapted with permission from ref (50). Copyright 2015 American Physical Society.

To try to better incorporate more of these correlation effects in noble metals, the adiabatic Gritsenko–van Leeuwen–van Lenthe–Baerends solid correlation potential (GLLB-SC) has been used to study Ag nanoparticles with RT-TDDFT, and the LSPR has been found to change significantly with system size. For icosahedral Ag nanoparticles ranging from 55 to 561 atoms, it has been found that Ag sp orbitals near the Fermi energy form a localized surface plasmon at opposing sides of the icosahedron, while d electrons polarize in the opposite direction, creating a screening field in the central region (Figure 2).^50^ Hence, accurate treatment of d-electrons is critical for reliably capturing plasmon behavior in Ag. It was found that individual single-electron transitions have a strong effect on the plasmonic response for the smallest nanoparticle (Ag_55_). However, for larger nanoparticles (Ag_147_ and above) the plasmon resonance is formed by constructive coupling of low-energy single-electron transitions. For these larger particles, the peak of the plasmonic response shifts to higher energies (i.e., blue-shifts) as the particle size decreases. A separate study was also able to discriminate differences between Ag_55_ and larger nanoparticles (up to 561 atoms) through the use of transition contribution maps. Ag_55_ excitations had strong contributions from individual Kohn–Sham transitions, indicating their single-particle character, while larger nanoparticles showed more transitions with off-resonant energies and a more continuous density of states, consistent with a stronger plasmonic character.^14^

**Figure 3:**
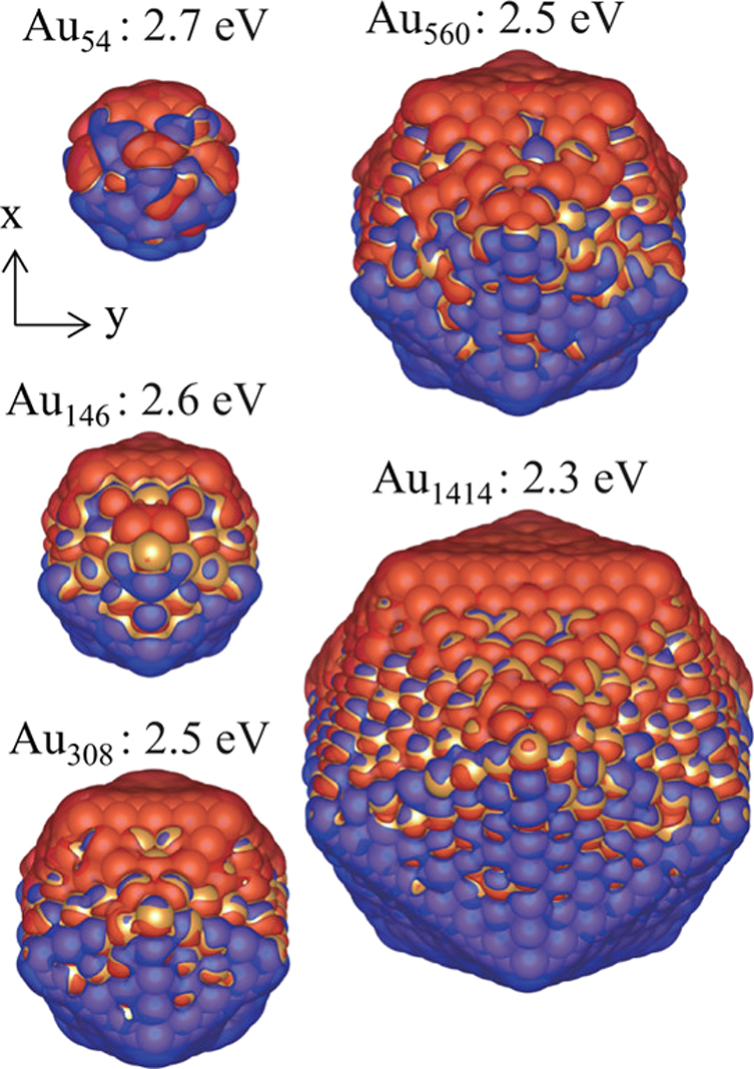
Induced charge densities (integrated over the entire propagation time of 30 fs) on Au_*n*_ (*n* = 54, 146, 308, 560, 1414). The density map thresholds are set to the same value, and the applied laser energies are shown above the nanoparticles. The charge density shows a clear separation as the nanoparticle size increases. Reprinted with permission from ref (56). Copyright 2014 American Chemical Society.

For icosahedral Au nanoparticles ranging from 54 to 1414 atoms, RT-TDDFT calculations have found that the LSPR peak shifts to higher energy with decreasing cluster size,^56^ similar to the results for Ag. However, at a sufficiently small cluster size (Au_54_ and Au_147_) the particles exhibit multiple resonance peaks and there is no clear LSPR peak due to a quantum confinement effect and the discrete energy levels that form at such a small size. As the cluster size increases, the separation of charge density becomes more clear (Figure 3). The charge densities around each Au atom inside the cluster were observed to oscillate in the opposite direction of the surface charge densities, consistent with a typical screening effect from d electrons in metal nanoparticles.^56^ The electron dynamics present in the inner region of the cluster are qualitatively similar to those discussed above for Ag.

As noted in some of the discussion above, sufficiently small systems (considered within the quantum regime) exhibit qualitative differences in plasmon generation such as multiple resonances. These differences were observed in Au spheres consisting of 68 to 600 electrons, which were simulated using a jellium-sphere model.^57^ The jellium model uses a uniform positive charge density instead of atomic potentials. This model does not allow for screening from core electrons. It was found that when the external field frequency was the same as the main peak frequency absorbed by the nanoparticle, a response is observed throughout the sphere, consistent with a classical surface plasmon. However, when driven at different external frequencies, the response occurs mostly near the center of the particle, referred to as “quantum core plasmons”. As the size of the sphere increases, the response of the classical surface plasmon becomes much larger than that of the quantum core plasmons. Multiple resonances were also found in the calculated absorption spectrum for another small system, the Ag_8_ tetrahedron.^18^ This spectrum showed sharp peaks at 3.05 and 3.96 eV. Even an icosahedral Ag_55_ nanoparticle showed qualitative differences from larger particles:^50^ Ag_55_ showed two broad peaks (Figure 2a), the largest around 4.2 eV, while larger particles exhibited a single broad peak around 3.6 to 3.8 eV.

Other systems such as chains and rings have been investigated for changes in the LSPR as the system size increases. In the chains, two plasmon modes exist (longitudinal and transverse), and it is often of interest to observe how these modes change with the number of atoms in the chain. The longitudinal mode is obtained by applying the external field in the same direction as the chain, while the transverse mode arises from an electric field applied perpendicular to the main axis.

For example, Na chains (up to *n* = 18) were investigated using RT-TDDFT with the LDA functional.^58^ It was found that the plasmon converges into a single resonance in the longitudinal mode but splits into two separate transverse modes (located at the ends of the chain and in the center). The splitting of the transverse mode was seen both in the optical absorption and in the induced electron density. The existence of two transverse plasmon modes was said to arise from both the variation of the electron potential on different atoms along the chain and interactions between electrons on different atoms. The splitting of the transverse mode into different spatial regions (which could in principle be controlled via either the chain length or applied external field) was postulated to allow for selectivity in chemical reactions of interest. In a separate study, Na rings with a thickness of either one or four atoms were investigated using the LDA functional.^59^ A weak δ-pulse (weak enough to remain in the linear-response regime) was applied along the nanoring and propagated using the Crank–Nicolson propagator. The one-atom thick ring showed a large absorption peak at 3.55 eV and three lower intensity peaks at lower energies. All peaks were found to be made of a large number of electron–hole transitions, indicating strong collectivity in the excitation. While this observation alone is not convincing evidence of the existence of a LSPR, the plasmonic nature of the ring is seen in the charge density visualization, in which a strong dipole is distributed continuously over the entire nanoring with positive and negative densities confined to the inner cavity and outer surface, respectively.

The dipole response of Cu, Ag, and Au chains up to 26 atoms was studied with the PW91 exchange-correlation functional, using a RT-TDDFT method which propagates the reduced single-electron density via the Liouville–von Neumann equation after an impulse excitation.^45^ The longitudinal mode (dominated by s → p transitions) displayed a red shift with increasing chain length, while the transverse mode (consisting of d → p transitions) was blue-shifted with increasing chain length. A different study of Ag chains, which used the LDA functional, found that the oscillator strength increased with increasing chain size and the resonance frequency red-shifted.^60^ In another study, Au chains (up to *n* = 12) were investigated using the BP86 functional.^17^ Several peaks were observed for the longitudinal mode, and these peaks both red-shifted and split with larger chain sizes. The splitting of these peaks was due to transitions from the d band. Conversely, the transverse mode remained relatively unaffected by the chain size, blue-shifting slightly. The longitudinal mode excitations were dominated by a single intraband transition while the transverse modes were made from a coupling of two or more single-particle transitions with delocalized Σ_n_ → Π_n_ character.

Few RT-TDDFT studies have done careful, consistent comparisons of multiple plasmonic metals. For Cu, Ag, and Au chains up to 26 atoms, clear differences in dipole response between the three metallic chains were observed, largely attributed to differences in energy levels. In Au_18_ and Cu_18_, the energy levels of s and d electrons are much closer than that of Ag_18_, which may result in stronger mixing of d electrons during the excitations in Au and Cu. Thus, the Ag chains are considered to be more free-electron-like than Au and Cu.^45^ In a separate study, absorption spectrum calculations of nanoparticles showed that Au_201_ and Cu_201_ lack the well-defined LSPR peak seen in Ag_201_ due to an earlier d-band onset in Au and Cu compared to Ag (2.1 and 2.3 eV for Au_201_ and Cu_201_, compared to 3.7 eV for Ag_201_).^61^

The above studies have revealed that the LSPR peak shifts to lower energies with increasing system size, in qualitative agreement with experimental studies.^62,63^ The separation of charge density and screening effect from d electrons can also be clearly seen with a growing cluster size. Further, systems in the quantum size regime show multiple resonances in their absorption spectra, likely due to the more molecular nature of their electronic structure; however, the plasmonic behavior is dominated by the classical surface plasmon as the particle size increases. In general, these particular quantum regime characteristics are observed in systems less than 55 atoms. The more classical picture of a surface plasmon arises for systems of several hundred atoms. In the small “quantum regime”, effects such as confinement, tunneling, and screening can all arise and should be considered.

## Mechanistic Studies of LSPR-Induced Reactions

A handful of studies have been performed to better understand the mechanism(s) behind adsorbate dissociation in plasmon-mediated reactions. Four mechanisms have been proposed and generally accepted to explain these types of photoreactions: (1) direct intramolecular excitation due to the near-field enhancement; (2) charge transfer between the metal and adsorbate states; (3) hot electron transfer from the metal to adsorbate; (4) local heating, in which the LSPR decay generates heat and induces a vibrational excitation. RT-TDDFT can be particularly insightful for these studies, as the reaction and excitation can both be observed in real time. However, determining causal relationships in these dynamic studies can be challenging; for example, just because charge transfer is observed does not necessarily mean it is important for driving dissociation. Thus, system parameters must be varied to try to gain these causal insights.

It is known that charge transfer is captured with varying degrees of accuracy depending on the functional used.^64−67^ As charge transfer is a nonlocal effect, GGA functionals have been seen to perform poorly in certain cases.^13,68^ In general, functionals which do not include exact exchange provide poorer estimates of most molecular system properties than hybrid functionals. This is well-known in molecular systems,^69^ but the implications for plasmonic systems are less clear. Further, at least in some systems, GGA orbitals do not hybridize to the same extent as LC orbitals. In cases where there is significant spatial overlap of the donor and acceptor, GGA functionals can be suitable for capturing charge transfer.^68^ Additionally, a few RT-TDDFT studies of plasmonic systems suggest that the choice of functional did not affect the overall physics or conclusions drawn.^22,70^

**Figure 4:**
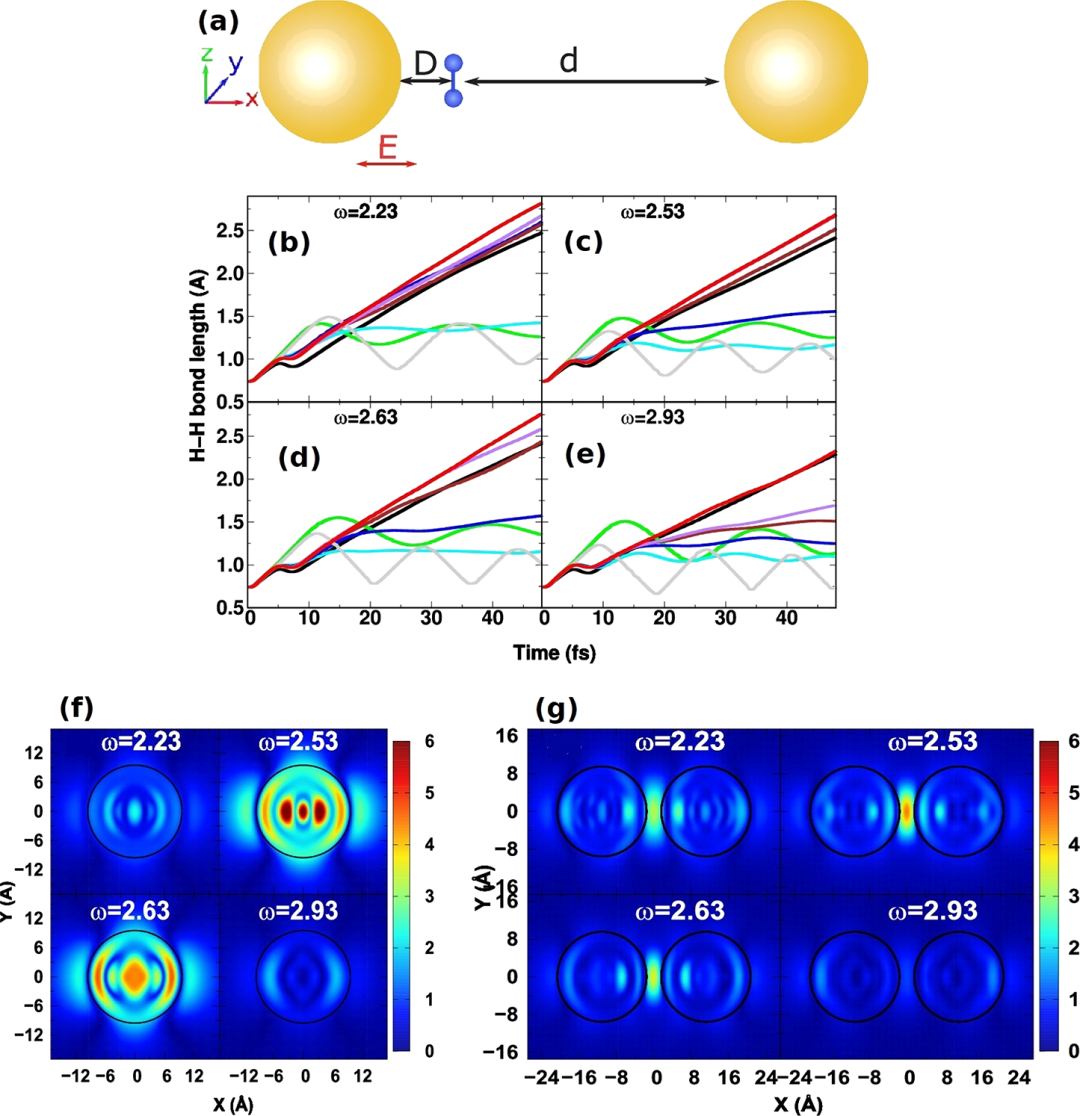
(a) Dimer arrangement of Au nanoparticles with H_2_ placed at a fixed distance *D* from the left nanoparticle and variable distances *d* from the right nanoparticle. (b–e) H_2_ bond lengths for a fixed *D* in the monomer (black) and dimer arrangement at *d* = 1.59 Å (green), *d* = 3.7 Å (cyan), *d* = 5.82 Å (blue), *d* = 7.94 Å (brown), *d* = 10.05 Å (purple), *d* = 12.17 Å (red), and *D* = *d* = 5.82 Å (gray). H_2_ dissociation is greatly reduced at *d* = 1.59 Å indicating that the chemical reaction can be suppressed or enhanced by adjusting the position of the molecule within the plasmonic dimer. The electric field enhancement is shown for the single nanoparticle (f) and the dimer (g). The dimer can generate enhancements in the gap which are larger than the field near the surface of the single nanoparticle. Adapted with permission from ref (22). Copyright 2018 American Chemical Society.

Small molecules such as H_2_, N_2_, and O_2_ are ideal model cases for studying the effect of charge transfer due to their simple electronic structure. Further, these molecules have relevance in a variety of chemical reactions such as ammonia synthesis, hydrogenation, and oxidation. H_2_ dissociation on a metallic Au nanoparticle with a diameter of approximately 19 Å was studied using RT-TDDFT with the LDA functional^22^ and the jellium approximation. Using just a single nanoparticle as well as a dimer of two nanoparticles, the induced charge, field enhancement, and H_2_ dissociation behavior were all investigated. The single nanoparticle enhanced the external field at both of its ends in the direction of the external field, while the dimer was found to generate “hot-spots” in the gap (Figure 4). Varying the placement of the H_2_ between the nanoparticles revealed that H_2_ dissociation could only proceed for certain distances between the particles; specifically, dissociation only occurred when there was significant charge accumulation on the H_2_. This result indicated the importance of charge transfer for H_2_ dissociation (via the hot electron mechanism) and the tunable nature of certain plasmonically driven reactions.

H_2_ dissociation on linear Ag chains (up to *n* = 12) has also been studied with RT-TDDFT and the LDA functional, resulting in the same conclusion that charge transfer is important for plasmon-mediated H_2_ dissociation.^60^ It was found that the charge density initially present in the H_2_ antibonding orbitals delocalizes over the H_2_ and Ag chain as time evolves. The significant overlap of H_2_ antibonding orbitals with Ag states allows for the external laser to drive charge to excited states and then couple to the antibonding H_2_ state, driving dissociation. In a separate study using the LC-ωPBE functional, H_2_ was found to activate readily even with small amounts of charge transfer from linear Ag chains.^71^

To gain mechanistic insight into plasmon-mediated N_2_ dissociation, RT-TDDFT was used with a variety of long-range corrected and GGA functionals to study the interaction of N_2_ with linear Ag chains (*n* = 4, 6, 8).^13^ It was found that N_2_ π* hybridization with Ag σ orbitals provided a path for plasmon-mediated charge transfer. Charge transfer to these N_2_ antibonding-like orbitals lead to activation of the N_2_ bond and ultimately, dissociation. In this case GGA functionals likely did not capture charge transfer between the two subsystems as accurately as the long-range corrected functionals. Relatedly, small Ag clusters and their interactions with N_2_ have been studied with the PBE0 functional over very short time scales to visualize the induced charge density under a weak field perturbation.^70^ There was a strong dependence on the orientation of the N_2_ of the energy flow between the molecule and cluster. In a separate study which used the LC-ωPBE functional, charge transfer was also found to be important for N_2_ dissociation on linear Ag chains. Compared to H_2_, N_2_ generally experienced a larger maximum charge change and did not activate as readily. Further, the maximum charge on H_2_ did not correlate as strongly with the degree of bond activation as the charge change for N_2_ did with its bond activation.^71^

To understand hot carrier transfer itself, 201-atom Ag, Au, and Cu nanoparticles interacting with CO were investigated with the GLLB-SC functional.^61^ It was found that the fraction of electrons generated in CO (after plasmon decay) did not decrease monotonically with increasing distance from the nanoparticle. Instead, more than one peak was observed for certain nanoparticle facets, suggesting that hot carrier charge transfer can be effective at relatively large distances and does not require molecular adsorption. Further, it was seen that the level alignment between molecular and nanoparticle electronic states is important for hot carrier transfer. These results indicate that ground state hybridization between molecular and metal states is a good predictor of hot carrier charge transfer and should be taken into account as a design principle. The importance of level alignment was also seen using a Ag_20_ nanocluster adsorbed on a TiO_2_(110) substrate using LDA. In this case, strong hybridization between Ag s and p states and the TiO_2_ conduction band allowed for charge injection from the Ag into the TiO_2_.^24^

**Figure 5:**
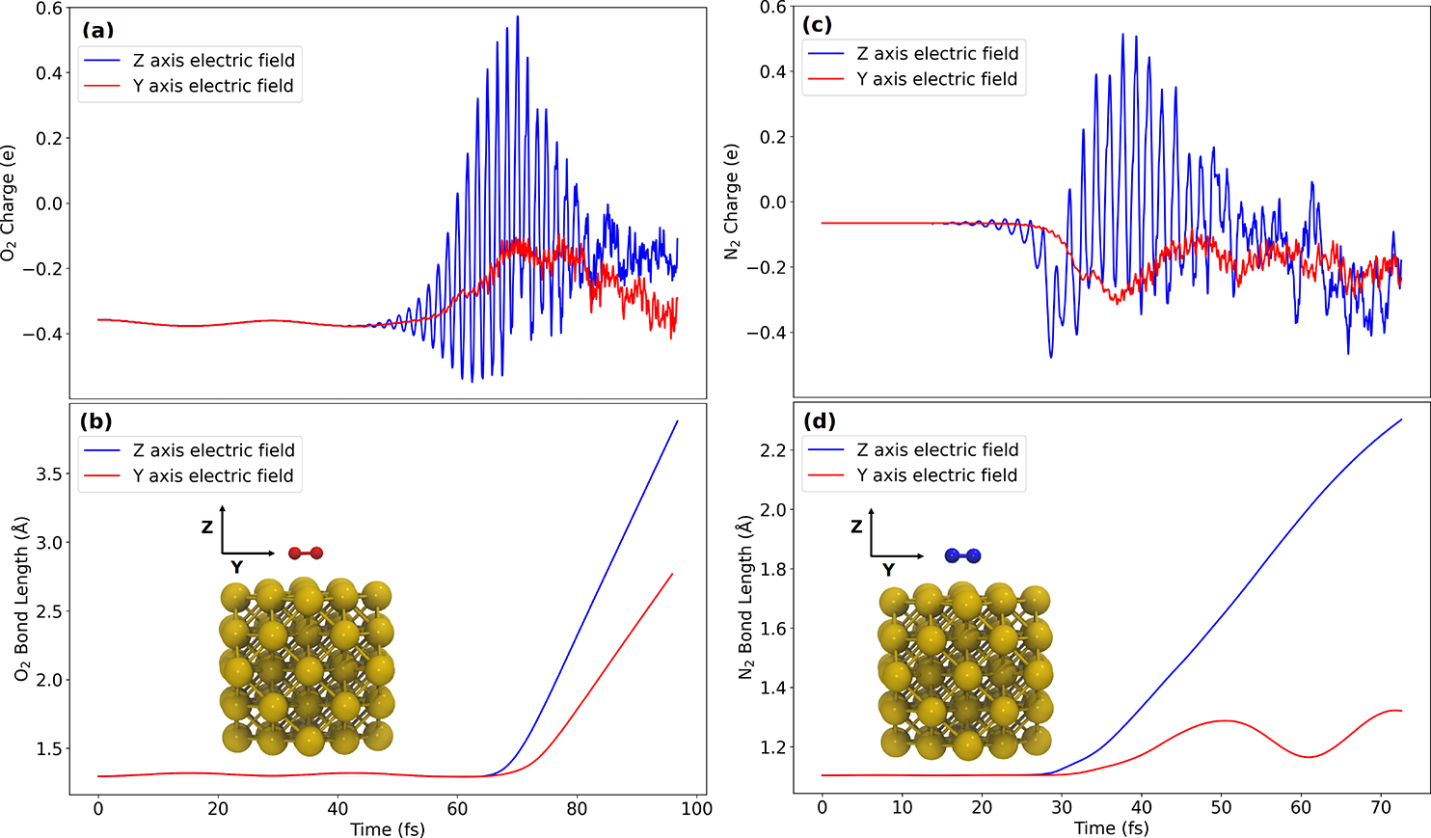
(a) O_2_ charge change and (b) bond length when subjected to electric fields polarized either along the molecular axis or normal to the Au_55_ nanoparticle surface. (c) N_2_ charge change and (d) bond length when subjected to electric fields polarized either along the molecular axis or normal to the Au_55_ nanoparticle surface. For O_2_, dissociation occurs in both cases while for N_2_ dissociation occurs only when the field is normal to the surface and large transient charge transfer is observed at roughly 25 to 30 fs.^72^ Reprinted with permission under a Creative Commons License from ref (72). Copyright 2023 the Authors, published by American Chemical Society.

Ag and Au nanoparticles of 19, 55, and 225 atoms were used to observe the dissociation of O_2_ and N_2_ at variable heights above the nanoparticles with the PBE functional. It was found that O_2_ dissociation could proceed with very minimal charge transfer while charge transfer was an important factor for N_2_ dissociation (Figure 5).^72^ In the case of O_2_, the near-field enhancement effect was the main driver of plasmon-mediated dissociation. Additionally, the electric field polarization could affect dissociation outcomes, further supporting previously discussed findings that the field polarization is important.

RT-TDDFT has also been used with the PBE functional to study the inverse but related process where a reaction induces excitations.^73^ It was found that when N_2_ or H_2_ dissociates on Ru nanoparticles, roughly half of the released energy initially generates electronic excitations, rather than phonons. It was also found that the dissociation barrier for N_2_ increases by approximately 0.2 eV due to nonadiabatic effects, and that the excitations from one dissociating N_2_ can affect another N_2_ on the same nanoparticle.

These studies show that RT-TDDFT can qualitatively reproduce the experimental finding that exposing plasmonic nanoparticles to light can significantly enhance some chemical reactions, such as molecular dissociation. Taken together, these studies suggest that charge transfer is important in plasmon-mediated dissociation of H_2_ and N_2_, while for O_2_ the near-field enhancement appears to be the sole mechanism. However, it is not yet clear how sensitive these results are to changes in the system and conditions. These studies also suggest that level alignment is likely important for allowing charge transfer. While quantitative interpretation of exact orbitals involved in these processes must be done with caution (due to the fictitious construction of Kohn–Sham DFT orbitals), it is reasonable to draw broader conclusions on the qualitative nature of the orbitals participating, a practice which is common in many types of DFT studies and is reasonably well supported.^74^

## Conclusions and Outlook

The studies presented here demonstrate the wide applicability of RT-TDDFT as a method for investigating plasmonic structures and their interactions with molecules. Generally, RT-TDDFT shows good agreement with the calculated excited states of LR-TDDFT, despite the lower computational cost for relatively large systems.

We have seen that accurate treatment of d electrons in metals is crucial for correctly capturing plasmon dynamics in transition metals and the screening effect they produce. Polarization of an external field is often employed and can lead to qualitatively different plasmonic behavior. Further, the size of the system will give rise to differences in the generated plasmon; most notably, a red shift in the LSPR mode occurs for increasingly large systems. Very small nanostructures show qualitatively different behavior from larger systems, such as multiple resonances and sharper peaks.

Both charge transfer and the near-field enhancement can facilitate dissociation of molecules, and the importance of these effects depends on the system, particularly the molecule. These mechanisms may allow for specific tuning of the system by altering the nanoparticle shape, size, and electronic structure.

There are many open questions related to the use of RT-TDDFT as a tool for studying plasmonic structures, with respect to both the computational methodology and the behavior of the systems. More comparison between RT-TDDFT and higher levels of theory, particularly for relatively realistic systems, would give stronger insight into the efficacy of RT-TDDFT and how its accuracy depends on the specific properties and systems under study. Additionally, further work that studies a variety of systems, with many careful control simulations to test various effects, will give insight into the generality of some of the conclusions that have been drawn, as well as changes across different systems. These broader studies could be particularly important for establishing general design principles and answering critical questions in plasmonic catalysis. For example, which mechanisms are important for a given system and can these mechanisms be predicted from the system’s ground-state electronic structure? Additionally, how do the various approximations made in current RT-TDDFT simulations impact charge transfer and other dynamics in the “high-field” regime? Finally, studies at longer time scales and of larger systems would allow clearer insight into how conclusions can be extrapolated to the field strengths, time scales, and length scales that are more relevant for experimental work.
